# Transport Mechanisms of Polymannuronic Acid and Polyguluronic Acid Across Caco-2 Cell Monolayers

**DOI:** 10.3390/pharmaceutics12020167

**Published:** 2020-02-17

**Authors:** Yu Wang, Xu Bai, Bo Hu, Maochen Xing, Qi Cao, Aiguo Ji, Shuliang Song

**Affiliations:** 1 Marine College, Shandong University, Weihai, Weihai 264209, China; wy392191187@163.com (Y.W.); 15634407267@163.com (X.B.); hobophar@163.com (B.H.); sddxxmc@163.com (M.X.); sddxcqq@163.com (Q.C.); jiaiguo@sdu.edu.cn (A.J.); 2 School of Pharmaceutical Sciences, Shandong University, Jinan 250012, China

**Keywords:** polymannuronic acid, polyguluronic acid, transport mechanisms, Caco-2 cell monolayers

## Abstract

Detailed knowledge of the intestinal transport of polymannuronic acid (PM) and polyguluronic acid (PG) is critical for understanding their biological activities. To investigate the transport in the gastrointestinal tract, PM and PG were chemically modified with tyramine and conjugated with fluorescein isothiocyanate (FITC) to synthesize FITC-PM (F-PM) and FITC-PG (F-PG) successfully. The transport mechanisms of F-PM and F-PG across the intestinal epithelial cell monolayers (Caco-2 cell monolayers) were then investigated. The results demonstrated that the transport of F-PM and F-PG into epithelial cells was time- and energy-dependent, which was mediated by the macropinocytosis pathway and the clathrin- and caveolae (or lipid raft)-mediated endocytic pathway. The transport process of F-PM and F-PG in Caco-2 cells depended on the acidification of endosomes and involved lysosomes. Tubulin mediated the transport of F-PM, but not of F-PG. Moreover, the absorption enhancer chitosan (CS) promoted the transport of F-PM and F-PG, increasing the apparent permeability coefficient (Papp) by 1.9-fold and 2.6-fold, respectively, by reversibly opening the tight junction (TJ). In summary, this study provided a comprehensive understanding of the transport of PM and PG in the small intestinal epithelial cells, which will provide a theoretical basis for the development of PM and PG with good intestinal absorption.

## 1. Introduction

Alginate is formed by co-polymerization of β-D-mannuronic acid (M) and α-l-guluronic acid (G) via a 1,4-glycosidic bond and can be degraded to obtain polymannuronic acid (PM) and polyguluronic acid (PG) [1]. As sodium alginate oligosaccharides, PM and PG have been reported to have antitumor, growth-promoting, antioxidant, and modulating intestinal microbial effects [2,3,4,5,6]. The activity of PM and PG is closely related to the mode of administration. The oral drug delivery system is the most widely used and most accessible manner among the various routes of administration, as it provides many advantages, such as being painless and easily self-administered [7]. Therefore, a detailed knowledge of the oral transport of PM and PG is important for its biological activities.

To date, few studies have reported the relevant transport mechanisms of PM and PG in intestinal epithelial cells. It has been widely recognized that the primary means of intestinal transport of drugs are the paracellular pathway and the transcellular pathway [8]. On the one hand, it is difficult for polysaccharide biomacromolecules to penetrate the tight junction (TJ) between cells due to their relatively high molecular weight (Mw), which severely limits their transport through the paracellular pathway [9]. On the other hand, transport of polysaccharide biomacromolecules through the transcellular pathway is complicated and involves endocytosis of polysaccharide molecules through the apical membrane, internalization in the cells, and ultimately exocytosis from the basolateral membrane [10]. Therefore, in-depth studies of the transport processes involved in the delivery of PM and PG through intestinal epithelial cells will provide some useful guidance for the development of PM and PG oral formulations with good intestinal absorption.

In this work, we first fluorescently labeled PM and PG and selected Caco-2 cells to mimic intestinal epithelial cell monolayers to study the intestinal transport mechanisms of PM and PG. We investigated the effects of the transport time, concentration, and temperature on the transport of PM and PG across Caco-2 cell monolayers. In addition, we used known inhibitors and siRNA techniques to study the transport mechanisms of PM and PG. These inhibitors used in our research were validated elsewhere. Finally, we also studied the effect of the absorption enhancer chitosan (CS), which acts by reversibly opening TJs between intestinal epithelial cells, on the transport of PM and PG. We hope that our research will provide a theoretical basis for the study of oral formulations of PM and PG.

## 2. Materials and Methods

### 2.1. Chemicals, Cells, Antibodies, and Small Interfering RNAs

Both PM and PG were obtained from Weihai International Biotechnology Research and Development Center with Mws of 15 kDa and 11 kDa, respectively. Tyramine, puromycin, sodium cyanoborohydride, fluorescein isothiocyanate (FITC), sodium fluorescein, chlorpromazine, and dynasore were purchased from Aladdin (Shanghai, China). Barbital sodium was purchased from JSKBIO (Qingdao, China). Methyl-β-cyclodextrin (MβCD) and MTT were purchased from Sigma-Aldrich (Saint Louis, MO, USA). Monensin and agarose were purchased from Solarbio (Beijing, China). Nocodazole, 5-(*N*-ethyl-*N*-isopropyl) amiloride (EIPA), and bafilomycin A1 were purchased from MCE (Princeton, NJ, USA). CS (Mw = 80 kD, degree of deacetylation ≥ 95%) was purchased from BOMEI (Anhui, china). FITC-transferrin was purchased from Jackson (Lancaster, PA, USA). FITC-dextran (10 kD) was purchased from TdB Consultancy (Uppsala, Sweden).

The human colon adenocarcinoma Caco-2 cell line was purchased from the Kunming Cell Bank of the Chinese Academy of Sciences (Kunming, China). Dulbecco’s modified Eagle’s medium (DMEM, high-glucose) was purchased from HyClone (Logan, UT, USA). Fetal bovine serum (FBS) was purchased from Zhejiang Tianhang Biotechnology (Zhejiang, China). Penicillin, streptomycin, and trypsin were obtained from Solarbio (Beijing, China). All other chemicals and solvents were analytical or chromatographic grade.

Rabbit anti-clathrin heavy chain monoclonal antibody (BST17625130) was purchased from BOSTER (Wuhan, China). Rabbit anti-caveolin-1 (AF1231), mouse anti-actin (AA128) monoclonal antibodies, HRP-conjugated goat anti-mouse IgG (A0216), LipoRNAi™ Transfection Reagent (C0535), and other reagents for the Western blotting experiments were purchased from Beyotime (Shanghai, China). Mouse anti-dynamin II monoclonal antibody (sc-166669), dynamin II siRNA (h) (sc-35236), and caveolin-1 siRNA (h) (sc-29241) were purchased from Santa Cruz (Dallas, Texas, USA). Rabbit anti-claudin-1(WL00448) and rabbit anti-occludin (WL01996) polyclonal antibodies were purchased from Wanlei Biotechnology (Shenyang, China). Rabbit anti-claudin-4 polyclonal antibody (ABP50989) was purchased from Abbkine (Wuhan, China). HRP-conjugated goat anti-rabbit IgG (ZB-2301) was purchased from ZSGB-BIO (Beijing, China). Clathrin heavy chain siRNA (h) was purchased from Sangon Biotech (Shanghai, China).

### 2.2. Fluorescent Labeling of PM and PG and Verification

PM and PG were fluorescently labeled according to previously described methods [11]. Briefly, 400 mg PM or PG were dissolved in 0.2 mol/L phosphate buffer (30 mL, pH = 8), followed by the addition of 400 mg tyramine and 150 mg sodium cyanoborohydride. The reaction was carried out at 37 °C for 96 h. Subsequently, the mixture was centrifuged (4000 r/min, 10 min) by centrifuge (Eppendorf, Hamburg, Germany), and absolute ethanol was added to the supernatant to a final concentration of 80% (*v/v*). The resulting precipitate was collected by centrifugation (4000 r/min, 10 min) and dissolved in 0.5 mol/L NaHCO_3_ (40 mL). Next, 25 mg of FITC were added, and the reaction was allowed to stand overnight in the dark at room temperature. The sample was desalted by Sephadex G-25 column (ρəе, Wuxi, China) and lyophilized to obtain FITC-PM (F-PM) and FITC-PG (F-PG).

The fluorescently labeled products were verified by agarose gel electrophoresis and fluorescence spectrophotometry [11]. For agarose gel electrophoresis, gelatin was prepared using a sodium barbital solution containing 0.5% (*m*/*v*) agarose. Three microliters (2 μg/μL) of FITC-labeled products were added to the tunnel. Electrophoresis was carried out, and the gel image was observed by a gel imager (Bio-Rad, Hercules, CA, USA). The same concentrations (500 μg/mL) of the samples were detected by a fluorescence spectrophotometer (PerkinElmer, Waltham, MA, USA). First, a full-wavelength scanning from 200–800 nm of each substance was performed, with the emission wavelength and the excitation wavelength slit width both set to 10 nm. The excitation wavelength was then set to the excitation wavelength of FITC (495 nm) and the difference in the emission spectra of each sample.

FITC-labeled and unlabeled samples and a series of known Mw dextran standards were passed through a TSK-gel G4000 PWxl column (TOSOH, Tokyo, Japan), equilibrated and eluted with 0.15 M NaCl, and passed through a 210 nm ultraviolet absorption curve to examine changes in Mw before and after labeling. Regression treatment was performed using logarithmic Mw (log Mw) of standard dextran versus retention time (t), and the Mw of each sample was calculated. Finally, we carried out experiments to determine the fluorescence labeling rates ($R_{L}$). FITC solutions with a certain concentration were prepared with Hanks’ Balanced Salt Solution (HBSS) solution, and HBSS was used as the reference solution. The fluorescence intensity of each standard solution was measured in turn using a multi-function microplate reader (Ex = 495 nm, Em = 515 nm, Bio-Tek, Winooski, Vermont, USA). Take the concentration of FITC as the abscissa and the fluorescence intensity as the ordinate, and then draw a standard curve. Several concentrations of FITC-labeled sample solutions were prepared using the HBSS solution. The fluorescence intensity value of each sample solution was measured according to the measurement method in the standard curve. The concentrations of the FITC group of the fluorescently labeled products were calculated from the standard curve, and $R_{L}$ was calculated according to the following formula:

(1) $$R_{L}\left(\%\right){\;=\;C}_{F}{/C}_{0}\;\times \;100\%$$

where $C_{F}$ is the concentration of the FITC group in the sample (μg/mL); $C_{0}$ is the initial concentration of the sample (μg/mL).

### 2.3. Establishment of the Caco-2 Cell Monolayer Model

Caco-2 cells were cultured in DMEM containing 20% (v/v) FBS, penicillin, and streptomycin (100 U/mL), and the cells were cultured in a 25 cm^2^ cassette culture flask and placed in a CO_2_ incubator (Thermo Electron Corporation, USA). When the cell density reached 80%-90% confluence, it was digested with 0.25% trypsin-0.02% EDTA and passaged at a ratio of 1:3. To investigate the transport mechanisms of PM and PG, we established a fast 7 day Caco-2 monolayer model based on the study by Sevin et al. [12]. Briefly, using 0.4 μg/mL puromycin instead of penicillin and streptomycin (100 U/mL), Caco-2 cells were seeded at a density of 2.5 × 10^5^ cells/mL (0.4 mL) in the upper chamber of Transwell devices (Costar Transwell, Millipore Corp. aperture: 0.4 μm, surface area: 0.336 cm ^2^). Add 1.0 mL of cell-free medium to the lower chamber of the Transwell. The liquid was changed every day, and a Caco-2 cell monolayer model was formed after 7 days of culture by using a Millicell-ERS voltammeter (Millipore Corporation, Billerica, MA, USA) to monitor the transmembrane resistance (Trans Epithelium Electrical Resistance, TEER). The TEER value was calculated according to the following formula:

(2) $${\mathrm{TEER}\;=(\mathrm{TEER}}_{T}{-\mathrm{TEER}}_{c})\times \;A$$

where TEER_T_ is the measured TEER value of the Transwell chamber with cells (Ω); TEER_C_ is the measured TEER value of the Transwell chamber without cells (Ω); A is the area of the Transwell chamber (cm^2^).

The TEER value of the Caco-2 cell monolayers exceeding >500 Ω·cm^2^ was considered to be qualified for subsequent transmembrane transport experiments. After modeling, 10 μg/mL of sodium fluorescein were added to the apical side to detect the permeability of the membrane (the specific method referred to Table 1). In addition, the medium of the apical and basal side was collected to detect its alkaline phosphatase activity by using an alkaline phosphatase kit (Njjcbio, Nanjing, China).

### 2.4. Cytotoxicity Tests

The cytotoxicity tests of Caco-2 cells were tested by the MTT assay. Briefly, 100 μL of cells containing 1 × 10^5^ cells/mL were seeded in 96 well plates, and after 24 h of culture, the cells were exposed to a series of concentrations of the samples at 37 °C for 24 h. The cells were then incubated with 20 μL of MTT solution (5 mg/mL) per well for 4 h at 37 °C. After the incubation, the culture medium in each well was replaced with 100 μL of DMSO to dissolve the blue (blue-violet) water-insoluble formazan crystals. Finally, the plate was shaken for 10 min at 37 °C, and the absorbance of each well was detected at 490 nm using a microplate reader (Tecan, Männedorf, Switzerland).

### 2.5. Transport Mechanisms of F-PM and F-PG

Caco-2 cells were seeded on Transwell polyester membranes and cultured for 7 days to form monolayers. The Caco-2 cell monolayers were washed three times with HBSS before each experiment and then incubated in a CO_2_ incubator for 30 min to reach equilibrium. We then studied the effects of different conditions on the transport mechanisms of F-PM and F-PG according to the methods described in Table 1.

The integrity of the cell monolayers was assessed by measuring the TEER value during experiments. After transporting F-PM and F-PG, the monolayer mold was cut out to wash, and the cells were lysed, then a Western blotting experiment was performed according to the method in “Section 2.8.” to check the integrity of the membrane. After the experiment, 200 μL of transport samples were collected from the basal side or the apical side, and the fluorescence intensity was measured with a multifunctional microplate reader (Ex = 495 nm, Em = 515 nm). The transport concentration of the sample was calculated according to the respective standard curve. The standard curve was obtained by the method shown in “Section 2.2.”. The apparent permeability coefficient (Papp) was calculated according to the following formula:

(3) $$P_{\mathrm{app}}{\;=\;(\mathrm{dQ}/\mathrm{dt})/\mathrm{AC}}_{0}$$

where dQ/dt is the transport of drug per unit time (mg/s); A is the area of the transport membrane (cm^2^); and $C_{0}\;$is the initial concentration of sample solutions (μg/mL).

The transmission rate ${(R}_{T})$ was calculated according to the following formula:

(4) $$R_{T}\;\left(\%\right){\;=\;(C}_{T}{\;\times \;V}_{T}{/(C}_{0}{\;\times \;V}_{0}))\;\times \;100\%,$$

where $C_{T}$ is the transport concentration (μg/mL); V_T_ is the transport volume of sample solutions (mL); $C_{0}$ is the initial concentration of the sample (μg/mL); V_0_ is the initial volume of the sample (mL).

The relative transport percentage (R_R_) was calculated according to the following formula:

(5) $$R_{R}\;\left(\%\right){\;=\;C}_{I}{/C}_{C}\;\times \;100\%,$$

where C_I_ is the transport concentration of the drug on the basal side after treatment with temperature, or inhibitor, or transfection (μg/mL); C_C_ is the transport concentration of the drug on the basal side in the control group (μg/mL).

### 2.6. Determination of Cholesterol Content

Caco-2 cells were seeded at 1×10^5^ cells/well in a 6 well culture plate and incubated for 4 days. A MβCD solution at the concentration indicated in Table 2 was added at 37 °C for 1 h, and then, the cells were washed three times with the HBSS solution. The cells were lysed by adding lysis buffer and centrifuged at 12,000 rpm for 10 min. The cholesterol content of the supernatant was quantified using a cholesterol analysis kit (Applygen, Beijing, China) according to the manufacturer’s instructions.

### 2.7. Effect of CS on TJ

Zero-point-four milliliters of 0.25% CS solution were added to the apical side for 2 h, and then, CS was removed. The apical side was washed three times, and HBSS solution was added for 12 h. TEER changes were monitored in real time throughout the experiment. At the same time, caco-2 cells were seeded at 1 × 10^5^ cells/well in a 6-well culture plate and incubated for 4 days. One milliliter of 0.25% CS was added at 37 °C for 2 h, and then, CS was removed. The cells were washed three times, and DMEM was added for different times. After washing the cells with PBS, Western blotting was used to investigate the effect of CS on TJ-associated proteins.

### 2.8. Western Blotting

Rapidly lysing the cells in radio immunoprecipitation assay (RIPA) buffer to collect the protein, the samples were boiled and normalized to equal concentrations. For protein separation, 10% polyacrylamide gels were run at 80 V for about 20 min and then 120 V until the loading buffer reached the bottom of the gel. Proteins were transferred to a nitrocellulose membrane (PVDF) (Bio-Rad, Hercules, CA, USA) using a wet blot transfer system (Bio-Rad Laboratories) for 1.5 h at 200 mA. After blocking for 2 h with 5% skim milk in tris-buffered saline with 0.05% Tween 20 (TBST), the membrane was incubated overnight at 4 °C with diluted primary antibody in blocking buffer. Subsequently, the membrane was washed three times using TBST and then exposed to a suitable alkaline phosphatase conjugated secondary antibody for 2 h at 37 °C and then visualized with ECL Plus enhanced chemiluminescence. Densitometric analysis of specific bands was performed using Image J software (National Institute of Health, Bethesda, MD, USA).

### 2.9. siRNA Transfection and Drug Uptake

Caco-2 cells were seeded at 8 × 10^4^ cells/well in a 24 well culture plate and incubated at 37 °C for 24 h prior to transfection. Cells at 70% confluency were transfected with clathrin siRNA, dynamin II (DNM2) siRNA, or caveolin-1(CAV1) siRNA in DMEM without serum for 72 h. Transfections were performed using LipoRNA™ according to the manufacturer’s instructions. The reduction in protein was estimated by Western blotting analysis. For the drug transport experiment, 0.5 mL of 50 μg/mL F-PM and F-PG were added to each well containing siRNA-transfected cells and incubated for 2 h. Next, the cells were washed three times with cold PBS. Then, the Caco-2 cells were lysed by adding 200 μL of cell lysis buffer for 30 min. The lysed cell solution was centrifuged at 4000 rpm for 10 min at 4 °C. The volume of supernatant and precipitate was increased to 1 mL by adding PBS and maintained at 50 °C for 30 min to dissolve F-PM and F-PG. Then, the mixture was centrifuged at 12,000 rpm for 10 min. The F-PM or F-PG content of each supernatant was measured using a multi-function microplate reader. The R_R_ (%) was calculated according to Formula (4).

### 2.10. Statistical Analysis

All data in the graphs were presented as the mean value ± standard deviation from three independent measurements. The statistical analysis was used in statistical software (SPSS, Chicago, Ill, USA) and GraphPad Prism 7.00 (GraphPad Software, Los Angeles, CA, USA). The statistical significance was assigned at *p* < 0.05.

## 3. Results

### 3.1. Fluorescent Labeling of PM and PG

**Figure 1:**
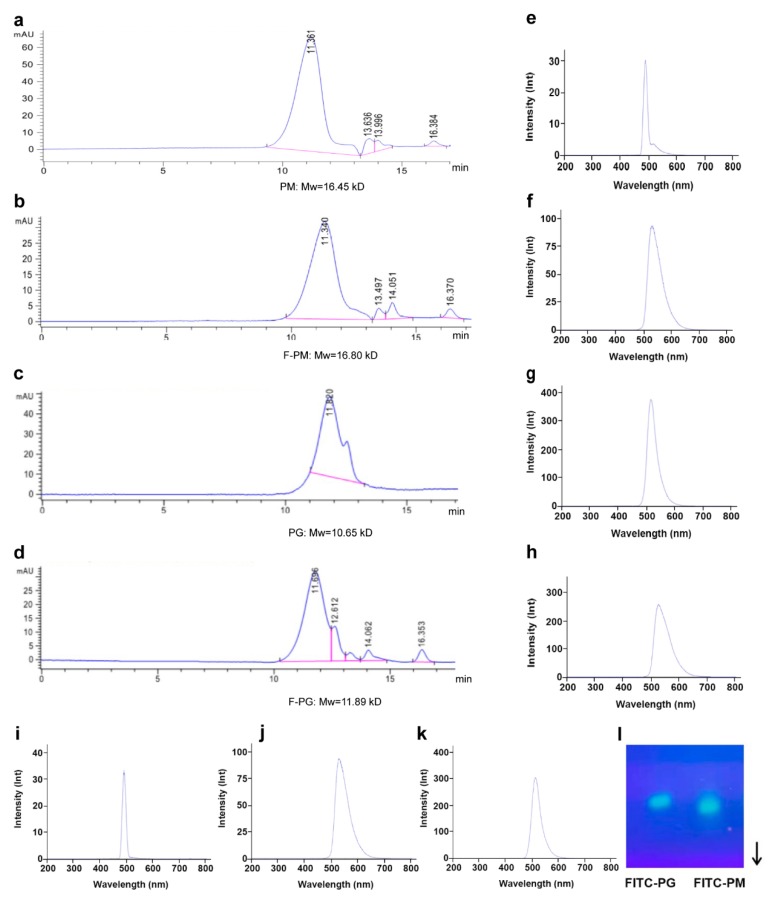
Mw determination by high performance gel permeation chromatography and verification of the fluorescent labeling of PM and PG by agarose gel electrophoresis and fluorescence spectrophotometry. Mw determination of PM (**a**), F-PM (**b**), PG (**c**), F-PG (**d**); fluorescence spectrum of unlabeled PM (**e**), a mixture of FITC and unlabeled PM (**f**), F-PM (**g**), FITC (**h**), PG (**i**), a mixture of FITC and unlabeled PG (**j**), and F-PG (**k**) at an excitation wavelength of 495 nm, lnt is the value of the fitted exponential function of the fluorescence intensity; (**l**) agarose gel electrophoresis pattern. The regression equation of dextran was *y* = −0.4207*x* + 8.9959 (R**^2^** = 0.9999).

We successfully synthesized F-PM and F-PG and verified the fluorescent labeling results by agarose gel electrophoresis and fluorescence spectrophotometry. The results of agarose gel electrophoresis (Figure 1l) showed that PM and PG each migrated as a single, bright green band, indicating their purity. The full-wavelength scanning results are shown in Table 3. The unlabeled PM and PG showed no emission wavelength, indicating the absence of fluorescence. The spectrum of FITC showed an excitation wavelength of 495 nm and an emission wavelength of 525 nm, which was close to the scanning spectrum of mixed solutions and the substance after labeling. Next, we set the excitation wavelength to 495 nm and scanned the fluorescence spectrum of the sample solutions. The results showed that unlabeled PM and PG had no emission wavelength peaks at 525 nm (Figure 1e,i). The FITC solution and the mixed sample solutions showed the same emission wavelength peak at 525 nm as the FITC solution. Since the excitation wavelength peaks of the three sample solutions at this time were close to the emission wavelength peaks, the excitation wavelength peaks were masked by the emission wavelength peaks, resulting in only a single peak (Figure 1f,h,j). Similarly, the FITC-labeled sample solution produced emission wavelength peaks near 525 nm, and a blue shift occurred in the chromatographic peak position (Figure 1g,k). The results of the Mw determination showed no significant difference in the Mws of PM and PG before and after labeling (Figure 1a–d). This indicated that tyramine and FITC had no effect on the Mws of PM and PG, which ensured the smoothness of subsequent experiments. We also measured the means of R_L_ of F-PM and F-PG, which were 12.66% and 9.52% (Table 4). As positive controls, we used commercial FITC-transferrin and FITC-dextran and measured the means of R_L_, which were 98.49% and 52.51%, consistent with their manufacturer’s instructions. In summary, the above results demonstrated that PM and PG were successfully labeled by FITC and could be used in subsequent experiments.

### 3.2. Establishment and Verification of Caco-2 Cell Monolayers Model

**Figure 2:**
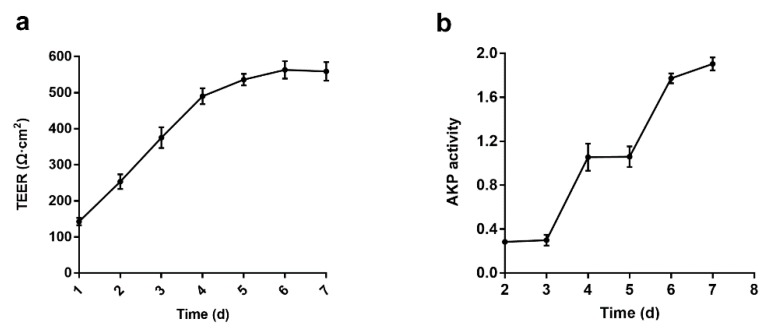
Validation of a fast seven day Caco-2 cell monolayer model. (**a**) Real-time monitoring of changes in TEER values; (**b**) alkaline phosphatase activity assay (AP/BL).

The Caco-2 cell monolayer model is widely used to mimic the absorption and transport of drugs across the intestinal epithelial layer [13]. In this paper, we used the method of Sevin et al. to establish a fast 7 day Caco-2 cell monolayer model to save modeling time. The addition of puromycin in DMEM resulted in the differentiation of Caco-2 cells, improved the barrier properties and purity of the cells, and increased the transcriptional expression level of p-glycoprotein [12,14,15]. There was a good correlation between the integrity of the Caco-2 cell monolayers and the TEER value [16]. As shown in Figure 2a, as the culture time increased, the TEER value increased rapidly and reached 500 Ω·cm^2^ on the fifth day. During the sixth and seventh days, it slowly further increased to 540 Ω·cm^2^ (>500 Ω·cm^2^), which proved that the Caco-2 cell monolayers were confluent and reached the experimental standard. Alkaline phosphatase (AKP) is a marker enzyme for intestinal epithelial cells, and its concentration is associated with the polarity and function of the Caco-2 cell monolayer [17]. Figure 2b shows that the ratio of apical to basolateral AKP activity (AP/BL) was 1.9 after seven days of culture, meeting the criteria for the Caco-2 cell monolayer model. In addition, the Papp of the osmotic marker sodium fluorescein was less than 1 × 10^−6^ cm/s at 30–240 min, and the R**_T_** (%) was very low, which also satisfied the standard of the Caco-2 cell monolayer model (Table 5).

### 3.3. Cytotoxicity of PM and PG

**Figure 3:**
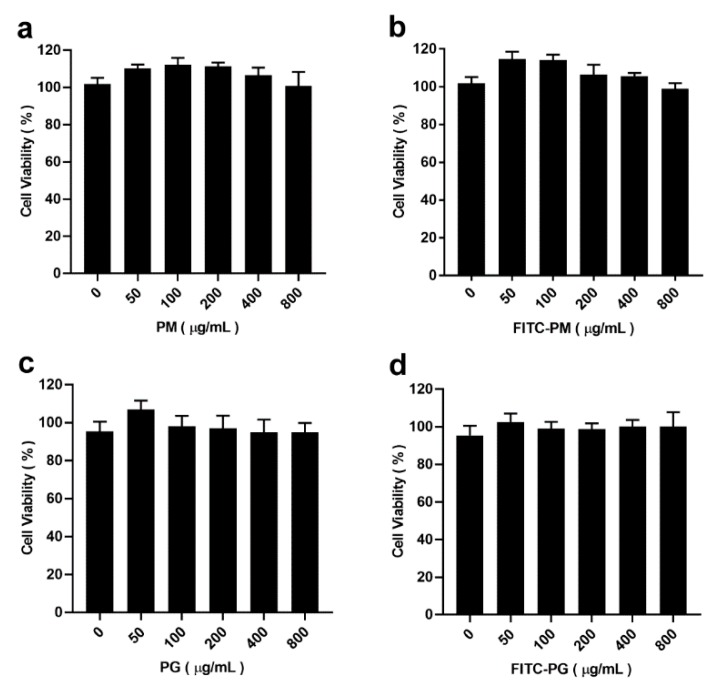
Lack of cytotoxicity of FITC-labeled and unlabeled PM and PG on Caco-2 cells. An MTT assay was used to test the cytotoxicity of PM (**a**), F-PM (**b**), PG (**c**), and F-PG (**d**) on Caco-2 cells.

Figure 3 shows the cytotoxic effects of PM, PG, F-PM, and F-PG on Caco-2 cells by an MTT assay. The results showed that, over the tested concentration range, PM and PG before and after labeling were not cytotoxic to Caco-2 cells within 24 h, indicating that they could be used in subsequent experiments.

### 3.4. Effects of Time, Concentration, and Temperature on the Transport of F-PM and F-PG

**Figure 4:**
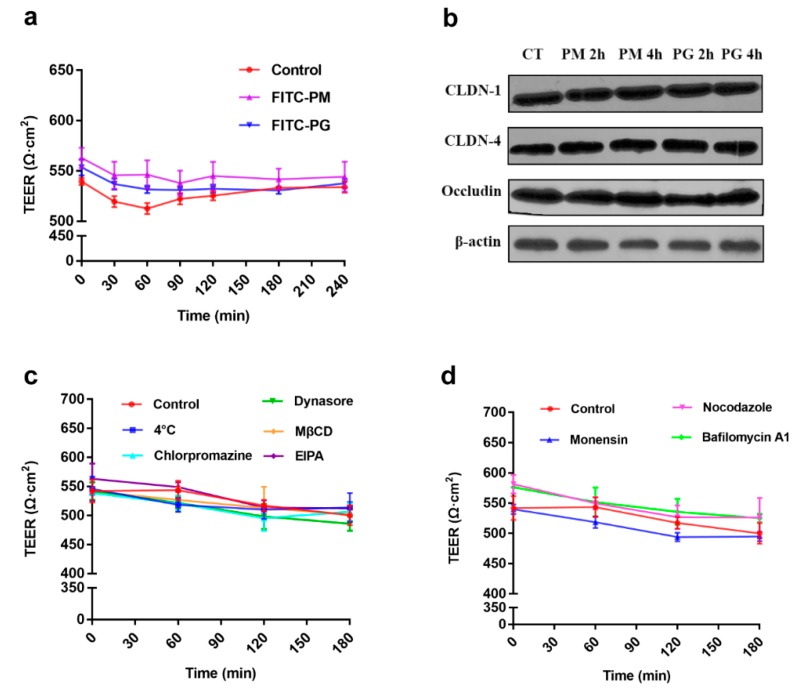
Transport-dependent changes in the TEER value and the impact of F-PM and F-PJ on tight junctions (TJ). (**a**) Effects of F-PM and F-PG on TEER during transport; (**b**) effects of F-PM and F-PG on tight junction proteins; (**c**) effects of the addition of transport inhibitors on TEER; (**d**) effects of the addition of intracellular inhibitors on TEER.

As reported, the molecular transport in the Caco-2 cell monolayers could be mediated by a transcellular or a paracellular pathway. For the paracellular pathway, TJs are required to open, and the TEER value is reduced during this process [18]. In our study, changes in TEER values were monitored in real time after adding the drugs. Figure 4a shows that the TEER values of the F-PM and F-PG groups were not significantly higher than the control group at any time point within 4 h, indicating that F-PM and F-PG did not enter Caco-2 cells via the paracellular pathway. This was consistent with the previously reported conclusion that the entry of biological macromolecular substances into the intestinal epithelial cells was restricted by TJs [19]. Table 6 shows the transport of F-PM and F-PG over different time intervals. In general, there was a good correlation between the Papp value of a drug in the in vitro Caco-2 cell monolayer model and its transportation in vivo. Drugs that can be transported in vivo typically display Papp values > 1 × 10^−6^ cm/s; on the contrary, Papp values < 1.0 × 10^−6^ cm/s indicate that the in vivo R**_T_** (%) was less than 1% [20]. As shown in Table 6, the Papp values of F-PM and F-PG were greater than 1 × 10^−6^ cm/s during the first 120 min of incubation, indicating that F-PM and F-PG could be transported orally. However, the Papp value was less than 1 × 10^−6^ cm/s at 240 min, indicating that F-PM and F-PG were poorly transported at this time. Since this may be related to the efflux of the drugs, we did a drug efflux experiment and found that F-PM and F-PG did not display efflux before 120 min, while after 120 min, the drug’s efflux rate increased, and after 240 min, the amount of efflux and the Papp value were close to the transportation (Table 7). Thus, we concluded that the reason why the transport of F-PM and F-PG appeared to come to a halt after 240 min was due to the efflux balancing the transportation. Table 8 shows the R**_T_** (%) of F-PM and F-PG at different concentrations. The transfer amount of F-PM and F-PG was constant regardless of the concentration, while their Papp values were negatively correlated with both transportation and concentration, which may be due to the limited number of sugar transporters. After completing the experiment, we lysed the cells on the membrane and extracted the protein to study the effect of F-PM and F-PG on TJ. It was found that both had no significant effect on TJ, and the TEER value did not change (Figure 4a,b). This ensured the integrity of the caco-2 cell monolayers upon completion of the experiment.

**Figure 5:**
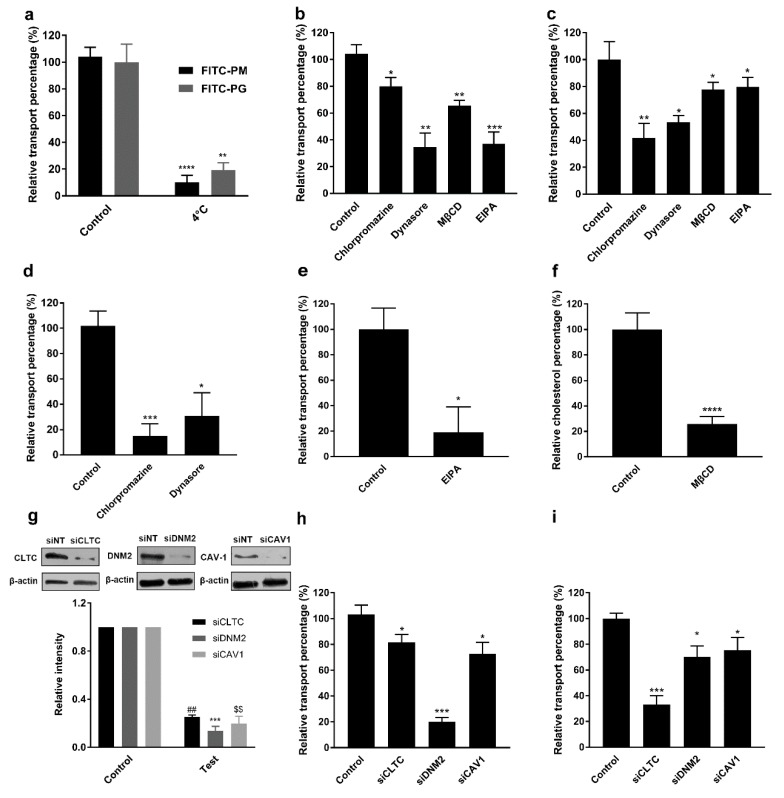
Effect of transport inhibitors and gene silencing on uptake of F-PM and F-PG into Caco-2 cells. (**a**) Effect of low temperature on the transport of F-PM and F-PG; (**b**) effect of transport inhibitors on F-PM transport; (**c**) effects of transport inhibitors on F-PG transport; (**d**) effects of chlorpromazine and dynasore on transferrin transport; the regression equation of FITC-transferrin was *y* = 952458*x* + 91,356 (R**^2^** = 0.9990, in the range of 0.001–6.25 μg/mL); (**e**) effect of EIPA on glucan transport; the regression equation of FITC- glucan was *y* = 512,495*x* + 35,382 (R**^2^** = 0.9999, in the range of 0.01–12.5 μg/mL); (**f**) effect of MβCD on total cholesterol in Caco-2 cells; (**g**) effect of siRNA silencing CLTC, DNM2, and CAV1. The values were normalized to a non-targeting siRNA (siNT) control; (**h**) the effect of gene silencing on F-PM transport; (**i**) the effect of gene silencing on F-PG transport. * *p* < 0.05; ** *p* < 0.01; *** *p* < 0.001; **** *p* < 0.0001; ## *p* < 0.01; $$ *p* < 0.01.

To investigate whether the transport of F-PM and F-PG was an energy-dependent process, we investigated the effect of temperature on the transport of F-PM and F-PG. The results showed that the R**_R_** (%) of F-PM and F-PG decreased by 90% and 81% at 4 °C compared with 37 °C, indicating that a low temperature could significantly inhibit their transport (Figure 5a) and that there was no change in TEER values under low temperature conditions (Figure 4c). In summary, the transport of F-PM and F-PG in Caco-2 cells was both time- and energy-dependent and was characterized by an efflux phenomenon.

### 3.5. Effects of Transport Inhibitors on the Transport of F-PM and F-PG

Since F-PM and F-PG entered the intestinal epithelial cells in a time- and energy-dependent process, we speculated that they entered the cells by endocytosis. Several pathways for endocytosis of polysaccharide biomacromolecules have been reported, including the macropinocytosis pathway, clathrin-mediated endocytic pathway, caveolae (or lipid raft)-mediated endocytic pathway, and clathrin/caveolae (or lipid raft) independent pathway [10,21]. To investigate the way F-PM and F-PG were transported into Caco-2 cells, we used several transport inhibitors. None of the inhibitors used in this study (Table 2) changed the permeability of the Caco-2 cell monolayers since they did not affect the TEER value (Figure 4c). This ensured the safety of all the inhibitors used in the transport experiments. EIPA can effectively inhibit the macropinocytosis pathway [22]. Chlorpromazine blocks the assembly of clathrin on the cell membrane, thereby inhibiting clathrin-mediated endocytic pathways [23]. Dynamin is required for clathrin and caveolae (or lipid raft), and dynasore can effectively inhibit the action of dynamin [24]. MβCD can bind to cholesterol, thereby inhibiting caveolae (or lipid raft)-mediated endocytic pathway [25]. The inhibition of the transport of transferrin by chlorpromazine and dynasore (Figure 5d) served as a positive control, since transferrin is often used as a marker for clathrin and caveolae (or lipid raft)-mediated endocytosis [26]. Likewise, the inhibition of glucan transport by EIPA also (Figure 5e) served as a positive control, while dextran was reported to act as a marker for the macropinocytosis pathway [27]. Concerning the effectiveness of MβCD in removing cellular cholesterol, quantitative analysis of cellular cholesterol after treatment of Caco-2 cells with MβCD for 1 h showed that MβCD could effectively remove intracellular cholesterol (Figure 5f), thereby inhibiting the caveolae (or lipid raft)-mediated endocytic pathway [26]. With the addition of chlorpromazine, dynasore, MβCD, or EIPA, the R**_R_** (%) of F-PM decreased by 19%, 65%, 34%, and 61%, and the R**_R_** (%) of F-PG decreased by 58%, 47%, 22%, and 20%, respectively (Figure 5b,c). However, some differences between F-PM and F-PG were observed. The reason for this different suppressive effect may be due to the different spatial structure of PM and PG. The structural differences between their structural units, mannuronic acid and guluronic acid, differed only at the hydroxyl position of C5, but when they were polymerized into chains, the spatial conformation was very different, which also caused the difference of their physical and chemical properties and biological activity. These results indicated that F-PM and F-PG entered Caco-2 cells via the macropinocytosis pathway and the clathrin and caveolae (or lipid raft)-mediated endocytosis pathway. This result was different from the transport mechanism of fucoidan that we reported previously, as the transport of fucoidan in the Caco-2 cell monolayers was almost completely blocked by chlorpromazine [11]. This confirmed that the absorption mechanism may be different for biomacromolecules with different structures.

To further validate the effects of the above inhibitors, we used siRNA technology and detected the silencing efficiency of siRNA by Western blotting [27,28]. The results showed that after silencing the clathrin heavy chain CLTC, DNM2, or CAV1, the silencing efficiency was greater than 85% (Figure 5g). The results of drug transport experiments after transfection showed that the R**_R_** (%) of F-PM decreased by 19%, 80%, and 27% and the R**_R_** (%) of F-PG decreased by 66%, 30%, and 24%, respectively, which further demonstrated that clathrin, dynamin II, and caveolin were essential for the entry of F-PM and F-PG into Caco-2 cells (Figure 5h,i).

### 3.6. Effect of Intracellular Inhibitors on the Transport of F-PM and F-PG

**Figure 6:**
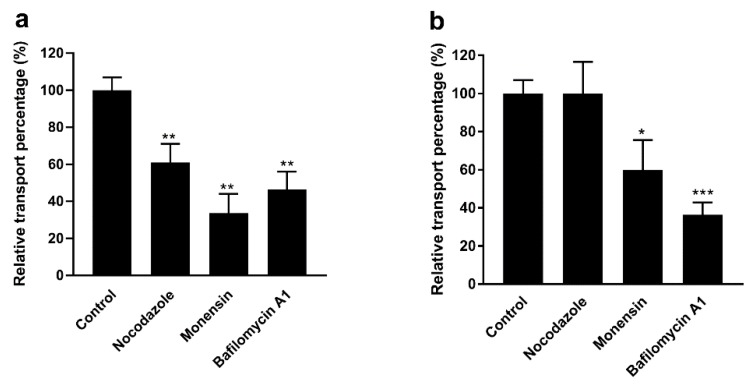
Uptake experiments of F-PM and F-PG after addition of intracellular inhibitors. (**a**) The effect of intracellular inhibitors on F-PM transport; (**b**) the effect of intracellular inhibitors on F-PG transport. * *p* < 0.05; ** *p* < 0.01; *** *p* < 0.001.

Some biomacromolecules are internalized by an endocytotic pathway. First, they are localized to the early endosomes, followed by transformation of early endosomes into late endosomes by endosome maturation, and finally, they are digested by lysosomes [27,29]. This process requires acidification of the endosomes. Here, we added various intracellular transport inhibitors to study the effect of endosomal maturation on the intracellular transport of F-PM and F-PG in Caco-2 cells. The intracellular transport inhibitors did not change the permeability of the Caco-2 cell monolayers since the TEER value was unchanged (Figure 4d). It has been reported that monensin inhibits endosome acidification and prevents endosomal maturation [30]. Bafilomycin A1, an inhibitor of the maturation process from early endosomes to mature lysozyme, was used to study the contribution of this maturation process to intracellular transport of F-PM and F-PG [31]. When these two inhibitors were added, the results showed that (Figure 6a,b) the R**_R_** (%) of F-PM was reduced by 66% and 54 and the R**_R_** (%) of F-PG was reduced by 40% and 64%, respectively. This indicated that the endosome maturation process was essential for the transport of F-PM and F-PG. Microtubules mediate the transport of many substances, and nocodazole is reported to inhibit tubulin-mediated transportation [32]. As shown in Figure 6a, the addition of nocodazole significantly reduced the transport of F-PM in Caco-2 cells (the R**_R_** (%) reduced by 39%), indicating that tubulin was also involved in the transport of F-PM in Caco-2 cells. However, nocodazole did not inhibit the translocation of F-PG (Figure 6b), indicating that tubulin may not be involved in the transport of F-PG. The reason for this difference may be due to the different spatial structure of PM and PG.

### 3.7. Effect of Absorption Enhancer on the Transport of F-PM and F-PG

Next, we investigated whether the transport of F-PM and F-PG was related to the opening of TJ between Caco-2 cells. The formation of TJ allows intracellular absorption of the required nutrients, ions, water, and electrolytes, but prevents pathogens, toxins, and other antigens from entering the systemic circulation [33,34,35]. Usually, TJs consist mainly of two types of proteins: transmembrane integrins (including claudins (CLDN), occludin, and linked adhesion molecules) and cytoplasmic scaffold proteins (ZO-1, ZO-2, and ZO-3). CLDN, occludin, and linked adhesion molecules, as well as a variety of regulatory proteins anchor the transmembrane protein to the actin cytoskeleton [8,35]. In fact, a TJ is not a static barrier, but a highly dynamic structure, so that it can be modified with a foreign substance such as an absorption enhancer, which is advantageous for promoting absorption of low-permeability drugs [36].

**Figure 7:**
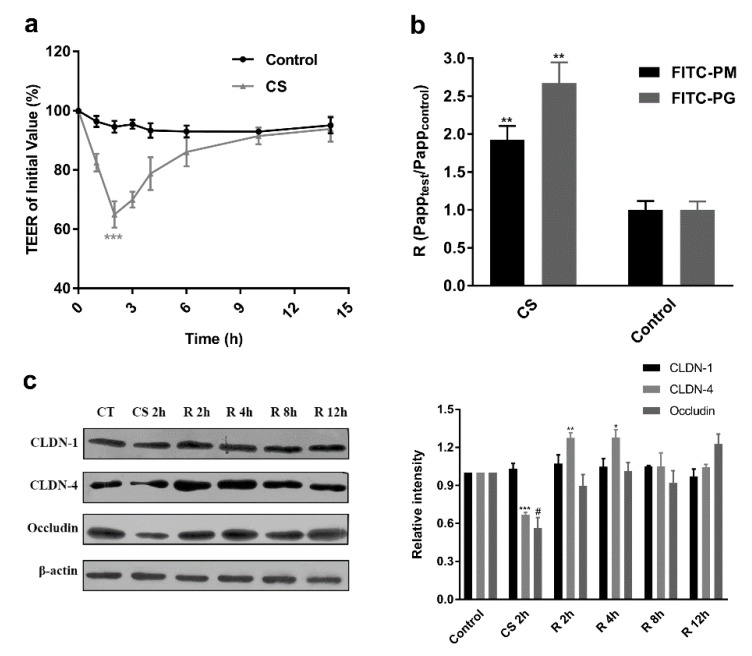
Effect of absorption enhancer on the transport of F-PM and F-PG in Caco-2 cell monolayers. (**a**) The effect of CS addition on TEER; (**b**) the effect of CS on F-PM and F-PG transport; (**c**) the effect of CS on tight junction proteins. * *p* < 0.05; ** *p* < 0.01; *** *p* < 0.001; # *p* < 0.05.

In order to increase the transport of F-PM and F-PG, we screened CS as a penetration enhancer. CS is widely considered to be a safe and effective intestinal absorption enhancer for therapeutic macromolecules due to its ability to reversibly compromise the integrity of the TJs of epithelial cells [37,38,39]. Therefore, we first studied the effect of CS on TEER values. Figure 7a shows that treatment of Caco-2 cells with CS for 2 h resulted in a significant decrease in TEER in the cell monolayer. Subsequent removal of CS that gradually restored TEER to its original level in 12 h. These results indicated that CS could induce the opening of TJs between Caco-2 cells and that the effect was reversible after CS was removed. Based on this conclusion, we then combined F-PM and F-PG with CS and found that the Papp values of F-PM and F-PG in CS-treated cells were 1.9-fold and 2.6-fold larger than those of the control group. Addition of CS significantly increased the translocation of F-PM and F-PG across the apical to basolateral direction of the Caco-2 cell monolayers at pH 6.5 (Figure 7b). According to several literature reports, the mechanism by which CS opens TJs relates to tight junction-associated proteins, so we examined the expression of tight junction-associated proteins (mainly CLDN1, CLDN4, and occludin) in Caco-2 cells after CS addition and removal. Cells were exposed to CS for 2 h, and expression of these proteins was detected using Western blotting. As shown in Figure 7c, the addition of CS resulted in a significant decrease in the expression of CLDN4 and occludin compared to control cells, whereas there was no significant difference in the expression of CLDN1. After removal of CS from the culture medium, the expression of occludin gradually returned to the initial level. In contrast, the expression of CLDN1 showed minimal change during cell recovery. Interestingly, the expression of CLDN4 increased during recovery (2 h and 4 h removal), which may be due to the fact that after the destruction of TJs following the addition of CS, a large amount of de novo synthesized CLDN4 was needed to compensate for this phenomenon. Our findings were consistent with those of Yeh et al. [40]. However, it should be noted that the mechanism by which CS promotes drug delivery may include many aspects. In addition, since the positive charge of CS may self-assemble with the negative anion compounds of PM or PG, further research about whether they will enter the intestinal epithelial cells in the form of endocytosis is needed. In our study, only the changes in expression levels of CLDN1, CLDN4, and occludin proteins upon the addition of CS were detected, but not the relocalization of these proteins. Previous research reported that CS-mediated TJ disruption was caused by translocation of TJ proteins from the membrane to the cytoskeleton. In intact TJ, transmembrane proteins were strongly associated with cell membranes. However, upon addition of CS, these proteins appeared to relocate from the membrane to other cellular compartments [40,41].

## 4. Conclusions

In recent years, much has been learned about the biological activities of sodium alginate-derived PM and PG, but there have been few studies on their intestinal transport. The molecular mechanisms that allow PM and PG to cross the intestinal epithelial cell monolayers are critical for the clinical application of low molecular sodium alginate. In this study, we found that the transport of F-PM and F-PG is a time- and energy-dependent process, while their transport capacity is very low (At 50 μg/mL, the R**_T_** (%) of PM and PG were only 1.71% and 1.14%). Different endocytic pathways mediate their transport processes, revealing that the pathways into the intestinal epithelial cells are different for different structural biomacromolecules. Our studies revealed that internalization of F-PM and F-PG was closely related to endosomal acidification, involved lysosomes, and that tubulin plays an important role in the transport of F-PM, but not of F-PG. This is the first study on the oral absorption mechanism of PM and PG, although there is no thorough study on the internalization of PM and PG in Caco-2 cells. We also studied the effect of the absorption enhancer CS on their transport rate. We found that CS can significantly increase the transport of F-PM and F-PG into the Caco-2 cell monolayer and that this mechanism of action is associated with changes in the expression of CLDN4 and occludin proteins. Our study thus provided a comprehensive understanding of the transport of PM and PG in the small intestine, which will provide some useful guidance for the development of PM and PG oral formulations with good intestinal absorption.

However, it should be noted that the oral transport mechanism of PM and PG provided by us needs further research, such as in vivo experiments. There should be the possibility that they are not just a transport mechanism. A recent article reported that PM can regulate the abundance of intestinal flora, and their transport mechanism may be related to the intestinal flora [2].

## Figures and Tables

**Table 1:** Effects of various factors on the transport of samples. EIPA, 5-(*N*-ethyl-*N*-isopropyl) amiloride.

Factors	Apical Side (0.4 mL)	Basal Side (1.0 mL)	Temperature (°C)	Transport Time (h)
**Transport**	F-PM/F-PG ^1^ (50 μg/mL)	HBSS	37	0.5, 1, 2, 4
**Efflux**	HBSS	F-PM/F-PG (50 μg/mL)	37	0.5, 1, 2, 4
**Concentration**	F-PM/F-PG (50,100,200,400,800 μg/mL)	HBSS	37	2
**Temperature**	F-PM/F-PG (50 μg/mL)	HBSS	4/37	2
**Inhibitors ^2^**	inhibitors for 1 h (remove) F-PM/F-PG (50 μg/mL)	HBSS	37	2
**Transferrin**	chlorpromazine or dynasore for 1 h FITC-transferrin (10 μg/mL)	HBSS	37	2
**Dextran**	EIPA for 1 h (remove) FITC-dextran (10 μg/mL)	HBSS	37	2
**Absorption enhancer**	0.25% CS (pH = 6.5) and F-PM/F-PG (50μg/mL) ^3^	HBSS	37	2
**Permeability**	sodium fluorescein (10 μg/mL)	HBSS	37	0.5, 1, 2, 3, 4

^1^ All sample solutions were prepared with HBSS. ^2^ For the investigation of the inhibitors, the cell monolayers were incubated with HBSS solution containing 0.4 mL of inhibitors (as shown in Table 2, including endocytosis inhibitors and intracellular transport inhibitors) for 1 h. After the incubation, the inhibitor was removed, and 0.4 mL of sample solution were added to the apical side. ^3^ Zero-point-four milliliters of a 0.25% CS (pH = 6.5) solution containing FITC polymannuronic acid (F-PM) or F-polyguluronic acid (PG) (the final concentration of F-PM or F-PG was 50 μg/mL).

**Table 2:** Functions and concentrations of transport inhibitors. MβCD, methyl-β-cyclodextrin.

Transport Inhibitors	Functions	Concentrations
**Endocytosis Process**		
chlorpromazine	inhibitor of clathrin-related route	20 μg/mL
dynasore	block the formation of a pinched-off vesicle	30 μg/mL
MβCD	caveolae (or lipid raft)-mediated route	2.5 mg/mL
EIPA	inhibit the macropinocytosis pathway	20 μg/mL
**Endocellular Transport Process**		
monensin	inhibit acidification of endosomes	30 μg/mL
nocodazole	inhibitor of microtubules	10 μg/mL
bafilomycin A1	inhibit the maturation process of lysosomes	100 μM

**Table 3:** Full wavelength scan value of the sample.

Conditions	Ex Max (nm)		Em Max (nm)	
	Wavelength	Intensity (lnt)	Wavelength	Intensity (lnt)
PM	329.0	7.823	-	-
PG	330.6	9.263	-	-
FITC	495.5	130.555	525.9	340.020
FITC and PM	512.5	140.311	529.0	399.075
FITC and PG	511.8	139.330	528.6	400.653
F-PM	494.0	63.748	514.6	385.438
F-PG	491.2	49.981	513.5	304.158

**Table 4:** The R_L_ (%) of F-PM and F-PG.

Sample	C_0_(μg/mL)	The Fluorescence Intensity	C_F_(μg/mL)	R_L_ (%)	The Mean of R_L_ (%)
**F-PM ^1^**	50	6,052,844	6.16	12.32	12.66
	25	3,138,633	3.15	12.62	
	12.5	1,660,217	1.63	13.05	
**F-PG**	50	4,699,162	4.77	9.53	9.52
	25	2,405,221	2.40	9.60	
	12.5	1,220,862	1.14	9.43	
**FITC-transferrin**	6.25	6,049,258	6.15	98.47	98.49
	3.12	3,026,260	3.04	97.25	
	1.56	1,589,678	1.56	99.76	
**FITC-dextran**	6.25	3,215,504	3.26	51.75	52.51
	3.12	1,648,774	1.60	51.83	
	1.56	895,269	0.84	53.96	

**^1^** The standard curve was obtained by plotting the fluorescence intensity on the ordinate and the concentration of FITC on the abscissa. The regression equation of FITC was *y* = 970,396*x* + 77,067 (R**^2^** = 0.9993, in the range of 0.003–6.25 μg/mL).

**Table 5:** Papp values and R_T_ (%) of sodium fluorescein at different times.

Time (min)	30	60	120	180	240
**Papp ^1^ (×10^−7^ cm/s)**	4.05 ± 0.742	1.83 ± 0.170	1.07 ± 0.268	0.79 ± 0.113	0.70 ± 0.028
**R_T_ (%)**	0.02 ± 0.004	0.05 ± 0.003	0.07 ± 0.009	0.10 ± 0.012	0.13 ± 0.013

**^1^** The regression equation of sodium fluorescein was *y* = 92,068*x* − 12,402 (R**^2^** = 0.9999, in the range of 0.1–12.5 ng/mL).

**Table 6:** Translocation of F-PM and F-PG at different times.

Time (min)	Transshipment at Different Times (ug/cm^2^)		Papp (×10^−6^ cm/s)	
	F-PM^1^	F-PG^2^	F-PM	F-PG
30	0.18 ± 0.050	0.12 ± 0.042	2.05 ± 0.559	1.32 ± 0.468
60	0.35 ± 0.004	0.20 ± 0.027	1.97 ± 0.022	1.22 ± 0.149
120	0.71 ± 0.047	0.44 ± 0.014	1.95 ± 0.153	1.21 ± 0.038
240	0.72 ± 0.039	0.53 ± 0.051	0.99 ± 0.054	0.74 ± 0.071

**^1^** The regression equation of F-PM was *y* = 121,734 *x* + 31,764 (R**^2^** = 0.9992, in the range of 0.025–50 μg/mL); **^2^** the regression equation of F-PG was *y* = 94,498 *x* + 3481.7 (R**^2^** = 0.9998, in the range of 0.025–50 μg/mL).

**Table 7:** The efflux experiments with F-PM and F-PG.

Time (min)	Transshipment at Different Times (μg/cm^2^)		Papp (×10^−6^ cm/s)	
	F-PM	F-PG	F-PM	F-PG
0–120	-	-	-	-
120	0.60 ± 0.002	0.33 ± 0.007	1.67 ± 0.005	0.91 ± 0.019
240	0.94 ± 0.251	0.59 ± 0.067	1.30 ± 0.035	0.83 ± 0.093

**Table 8:** Translocation of F-PM and F-PG at different concentrations.

Concentration (μg/mL)	Transport Concentration (μg/mL)		Papp (×10^−6^ cm/s)		R_T_ (%)	
	F-PM	F-PG	F-PM	F-PG	F-PM	F-PG
50	0.26 ± 0.031	0.16 ± 0.033	2.12 ± 0.260	1.31 ± 0.276	1.71 ± 0.210	1.14 ± 0.111
100	0.29 ± 0.061	0.15 ± 0.044	1.21 ± 0.253	0.63 ± 0.184	1.04 ± 0.101	0.56 ± 0.074
200	0.25 ± 0.042	0.12 ± 0.006	0.52 ± 0.086	0.24 ± 0.013	0.44 ± 0.035	0.20 ± 0.005
400	0.23 ± 0.019	0.14 ± 0.031	0.24 ± 0.020	0.15 ± 0.032	0.20 ± 0.008	0.13 ± 0.013
800	0.26 ± 0.065	0.13 ± 0.014	0.13 ± 0.033	0.07 ± 0.007	0.12 ± 0.014	0.06 ± 0.003
